# Distribution of cause of death in rural Bangladesh during 2003–2010: evidence from two rural areas within Matlab Health and Demographic Surveillance site

**DOI:** 10.3402/gha.v7.25510

**Published:** 2014-10-29

**Authors:** Nurul Alam, Hafizur R. Chowdhury, Ali Ahmed, Mahfuzur Rahman, P. Kim Streatfield

**Affiliations:** 1 Centre for Population, Urbanization and Climate Change, International Centre for Diarrhoeal Disease Research (icddr,b), Dhaka, Bangladesh; 2 Formerly with Health Information System Knowledge Hub, School of Public Health, University of Queensland, Brisbane, Australia

**Keywords:** InterVA, verbal autopsy, cause of death, Matlab, Bangladesh

## Abstract

**Objective:**

This study used the InterVA-4 computerised model to assign probable cause of death (CoD) to verbal autopsies (VAs) generated from two rural areas, with a difference in health service provision, within the Matlab Health and Demographic Surveillance site (HDSS). This study aimed to compare CoD by gender, as well as discussing possible factors which could influence differences in the distribution of CoD between the two areas.

**Design:**

Data for this study came from the Matlab the HDSS maintained by the International Centre for Diarrhoeal Disease Research, Bangladesh (icddr,b) since 1966. In late 1977, icddr,b divided HDSS and implemented a high-quality maternal, newborn and child health and family planning (MNCH-FP) services project in one half, called the icddr,b service area (SA), in addition to the usual public and private MNCH-FP services that serve the other half, called the government SA. HDSS field workers registered 12,144 deaths during 2003–2010, and trained interviewers obtained VA for 98.9% of them. The probabilistic model InterVA-4 probabilistic model (version 4.02) was used to derive probable CoD from VA symptoms. Cause-specific mortality rates and fractions were compared across gender and areas. Appropriate statistical tests were applied for significance testing.

**Results:**

Mortality rates due to neonatal causes and communicable diseases (CDs) were lower in the icddr,b SA than in the government SA, where mortality rates due to non-communicable diseases (NCDs) were lower. Cause-specific mortality fractions (CSMFs) due to CDs (23.2% versus 18.8%) and neonatal causes (7.4% versus 6%) were higher in the government SA, whereas CSMFs due to NCDs were higher (58.2% versus 50.7%) in the icddr,b SA. The rank-order of CSMFs by age group showed marked variations, the largest category being acute respiratory infection/pneumonia in infancy, injury in 1–4 and 5–14 years, neoplasms in 15–49 and 50–64 years, and stroke in 65+ years.

**Conclusions:**

Automated InterVA-4 coding of VA to determine probable CoD revealed the difference in the structure of CoD between areas with prominence of NCDs in both areas. Such information can help local planning of health services for prevention and management of disease burden.

Bangladesh is the most densely populated and the eighth most populous country in the world (1). Despite its widespread poverty, turbulent past, and occasionally extreme climate events, the country has made remarkable progress in the last four decades in providing vaccines to children and mothers; reducing vitamin A deficiency; lowering fertility, as well as mortality of infants, children and mothers; increasing life expectancy; and reducing gender and economic inequalities in health outcomes (2, 3). Since independence in 1971 the government has been pursuing a policy of health development that ensures provision of basic services to the entire population, particularly to the underserved population in rural areas. Within the overall development policy framework community-based approaches in health service delivery; partnership of the public sector with non-government organizations (NGOs) to reach deprived populations and address service gaps; and rapid adoption of context specific innovative technologies and policies have been instrumental in improving the health service coverage and health outcomes (4). All of these are accelerating the demographic and epidemiologic transitions, and the country now faces new population and health challenges as the burden of infectious diseases has shifted towards non-communicable diseases (NCDs) in the last decades (5, 6).

The demographic and epidemiologic transitions are likely to lower not only the overall risk of dying, but also the risk of dying from certain diseases more than others. The distribution of the risk factors and lifestyle can, however, change the structure of cause of death (CoD), but there is little empirical evidence due to a lack of reliable data. Introduction of a community-based integrated maternal, newborn and child health, and family planning (MNCH-FP) services project by icddr,b in late 1977 in half of the Matlab Health and Demographic Surveillance site (HDSS) that has been functioning since 1966, resulted in differences in the fertility and mortality (infant, child, and maternal) rates between the icddr,b service area (SA) and the government SA during 1978–2009 (7–9). The majority of the differences in the health outcomes between the two areas of comparable socioeconomic and demographic conditions were due to the differences in the quality of MNCH-FP services (10, 11). The differences in the fertility and mortality rates in these two areas for a longer period may have changed the structure of CoD, but this has not been examined systematically. Introduction of the WHO verbal autopsy (VA) into the HDSS in 2003 and development of the computerized automated algorithms for processing VA symptoms to reliably determine probable CoDs, provide a unique opportunity to compare CoD between the two rural areas with a different MNCH-FP services delivery provision within the Matlab HDSS during 2003–2010. This study aimed to exploit this opportunity using the HDSS mortality and VA data and compare CoD across gender in these two rural areas. The study results may help planning of health services for areas with reference to the local health service delivery system in order to prioritise disease burden considering local sociodemographic conditions.

## Methods

### The study site and local health service delivery system

The study site is Matlab, a sub-district of Chandpur district, where icddr,b maintains a HDSS since 1966. Matlab is 55 km southwest of the capital city Dhaka, Bangladesh. The government of Bangladesh provides primary health care services for a nominal fee through a three-tiered health service delivery system at the sub-district level: community clinics, each serving about 6,000 people, the health and family welfare centres, each serving 25,000 people, and the Upazila (sub-district) health complexes, each serving 250,000 people. The Upazila Health Complex situated at Upazila headquarters coordinates services between different tiers.

**Fig. 1:**
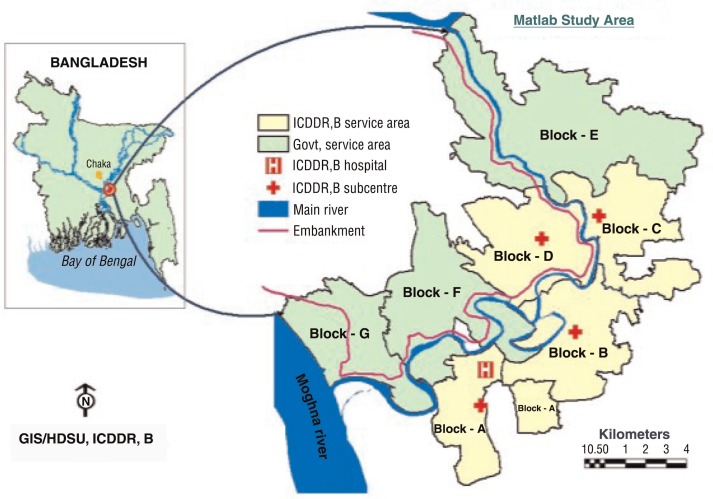
Map of Matlab demographic surveillance area showing icddr,b (yellow) and the government (green) service areas.

In late 1977, the icddr,b divided the HDSS into an icddr,b SA and a government SA (Fig. 1) and implemented a community-based MNCH-FP services project in the icddr,b SA in addition to usual public and private MNCH-FP services that serve the government SA. The icddr,b has set up at Matlab Upazila headquarters a primary care hospital (70 beds for managing patients with diarrhoeal diseases coming from anywhere and 60 beds for managing patients with maternal, newborn, and child health problems coming from the icddr,b SA only) and four community-based treatment centres in support of the hospital, which provide health services to women of reproductive age and children under 5 years old. These primary care hospital and four community-based treatment centres are located within the icddr,b SA. Over the years new service components complimentary to the MNCH-FP services were added in phases. The 50-bed public Upazila Health Complex with provision for outpatient and inpatient care facilities, and an operation theatre for minor surgery and caesarean section is also located at Matlab headquarters and falls within the icddr,b SA. Also more private fee-for-service clinics and unqualified health care providers (i.e. village doctors, drug sellers, homeopaths, and herbalists) are available in the icddr,b SA. The icddr,b primary care hospital and the four treatment centres are better equipped compared to public health facilities in terms of staff attendance, supervision, supply of medicines and logistics, quality of care, and effective referral service (10, 11). The icddr,b SA has greatly enhanced quality of health care services compared to the Government SA.

### Identification of deaths and training of VA interviewers for VA collection

Female community health research workers (CHRWs) having at least 10th grade education, visited households monthly up to 2006 and bi-monthly since then to record health and demographic events: births, deaths, migrations, marriages, and marital disruptions. Households with deaths registered by CHRWs during routine household visits have been visited by Field Research Supervisors (FRS) with WHO standard VA questionnaires since 2003. VA refers to interviewing close family members of the deceased about the events surrounding the fatal illness episodes or conditions. It contains both open narratives related to death and close-ended leading questions to elicit symptoms and signs of illness or conditions leading to death. For neonatal deaths, it contains a description of the mother's pregnancy and delivery care. The VA questionnaires in English were customized to suit local conditions, by reducing number of questions on HIV/AIDs and malaria, as prevalence of these diseases in the HDSS is very low, and adding few additional questions on arsenic related disease symptoms because ground water in the HDSS is arsenic contaminated. The customized VA questionnaires were translated into Bangla for training in the field data collection.

A public health physician and a medical demographer provided 4 days training to FRSs of non-medical background and a field research officer (FRO with 3 years training in medicine) on VA questionnaires, followed by 2 days of field practice. FRSs interviewed, with the VA questionnaires, the closest caretakers/relatives who had lived with the deceased in the same household around the time of terminal illness or death, within 6–12 weeks after the date of death. The FRO regularly supervised the fieldwork and the public health physician provided technical support in terms of clarification of the questions or disease symptoms when required. Informed standard consent forms were used to inform the respondents about the purpose of the study and guarantee confidentiality of information provided. Willingness to take part was expressed by signature or thumb impression. The Matlab HDSS data collection is approved by the Ethical Review Committee of icddr,b.

### Quality control

Scheduled revisits to 5% of randomly selected VA questionnaires were part of the quality control measures. The FRO visited the FRSs during data collection and reviewed the surveillance data collected at the household level, including VA filled-in questionnaires. Immediate feedback was provided. The FRO then completed mandatory checks and edited all filled-in VA questionnaires before sending them for coding and computer entry.

### Assessment of CoD from VA

The computer automated models are increasingly used for analysing VA symptom data to derive probable CoD, for being fast, low-cost and reliable compared to the physician-coded CoD (12–14). These models processed a range of items of VA information about the background characteristics and circumstances of a death, details of any illness (signs and symptoms) or conditions leading to death, previous medical history, etc. in a mathematical model based on Bayes’ theorem, and produced likely cause(s) of death (15, 16). The signs and symptoms or conditions, alone or in combinations, are highly indicative of specific diseases and infer the ‘most likely’ biomedical cause(s). VA data on symptoms and signs of illness or conditions that led to death collected under earlier standards of VA were converted to the WHO 2012 standard (17). The probabilistic model InterVA-4 (version 4.02) with options of low HIV/AIDS and malaria in the HDSS was applied to the individual VA information to assign probable CoD categories as defined by the WHO 2012 VA standards. The InterVA-4 yields, for each case, up to three possible CoD or an indeterminate result. It thus gives, if not indeterminate, the most likely CoD with an estimated probability for this cause. If the sum of the estimated probabilities for the first, second, and third most likely causes of death was less than 100%, the residual component was then assigned as being indeterminate.

### VA data analysis

Matlab HDSS provided VA data generated during 2003–2010 to the INDEPTH multisite dataset (18). Of 12,144 deaths that occurred in the HDSS area, VA symptom data could not be obtained for 129 deaths: 31 cases for non-response and 98 cases for no symptoms or signs of illness prior to death. The InterVA-4 coded CoD were then broadly grouped into communicable diseases (CDs), NCDs, perinatal and neonatal causes, pregnancy-related deaths, injury and other external causes, or indeterminate. Estimated broad cause-specific mortality rates for children (<15 years), adults (aged 15–64 years) and elderly were compared between the two areas. Also estimated cause-specific mortality fractions (CSMFs) per 100 deaths by sex within the areas were used to examine differentials in burden of disease. The difference between the two death rates was compared using Chi-square test and the difference between the two fractions was compared by Z-test for statistical significance at 80% power and p<0.05.

## Results

### Demographic, social, and health profiles in two service areas

Age distributions (in%), mean ages, and sex ratios of the populations in the two adjacent areas in 2010 were comparable (Table 1). Social indicators, such as level of education of youth aged 15–24 years, are comparable between the two areas. Although total fertility rates since 2005 were comparable between the two areas, the infant and child mortality rates were lower in the icddr,b SA. Coverage of immunizations of children and mothers with tetanus toxoid were very high in both areas, but the antenatal care coverage, facility-based delivery, caesarean sections, pneumonia treatment from a well-trained provider, and management of diarrhoea with oral rehydration solution and with zinc tablets were very low in the government SA. Both the areas were similar in demographic and social indicators, but different in health indicators.

**Table 1:** Demographic, social and health indicators in the icddr,b and government service areas, Matlab HDSS 2010

Demographic, social and health indicators	icddr,b service area	Government service area
Mean age±standard deviation		
Male	28.2±21.4	27.7±21.3
Female	28.8±20.3	29.0±20.6
Sex ratio of males per 100 females	87.2	86.5
Age distribution (in %) of the population:		
Children aged <15 years (%)	32.8	33.4
Adults aged 15–64 years (%)	60.6	59.7
Elderly aged 65+years (%)	6.5	6.8
Education of youth aged 15–24 years in 2013:		
None (%)	3.2	3.6
Primary (up to class V) (%)	18.0	20.3
Secondary & above (class VI+) (%)	78.8	76.1
Total fertility rate per woman	2.6	2.5
Infant mortality rate per 1000 live births	25.1	35.4
Child (1–4yrs) mortality rate per 1000 children	2.1	2.5
Crude death rate per 1000 populationa^a^	6.7	6.7
Coverage of full immunization:		
Children aged 12–23 months (%)	88.6	87.4
Mother with tetanus toxoid (%)	97.0	86.4
Antenatal care received from skilled providers:		
1^st^ trimester (%)	47.9	12.0
2^nd^ trimester (%)	96.2	60.4
3^rd^ trimester (%)	97.5	78.7
Delivery in a health facility (%)	80.7	28.1
C- section (%)	22.8	15.2
Seeking treatment from trained health providers:		
for ARI/pneumonia (%)	39.8	31.8
Management of diarrhoeal episode:		
with oral rehydration solution (ORS) (%)	36.6	24.0
ORS+zinc tablet (%)	17.2	10.6

Source: Matlab HDSS annual reports published in 2012 and 2014, icddr,b, Dhaka, Bangladesh.

a The rate was lower in the icddr,b SA than the government SA for in last 10 years except in 2010.

### Mortality rates in 2003–2010 by area

**Fig. 2:**
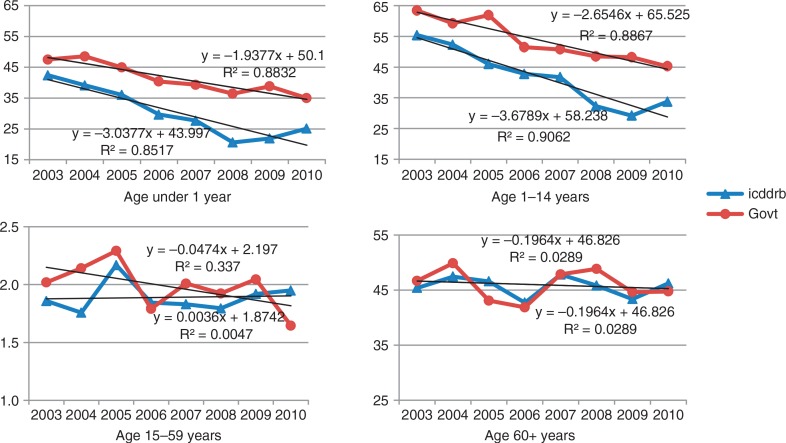
Trends in mortality (per 1,000 person-years) in difference age groups by area.

The HDSS recorded 5,965 deaths in the icddr,b SA and 6,179 in the government SA during 2003–2010. Yearly death rates by age and area showed declining trends in infant and child (age 1–14 years) mortality rates in both areas and the declines were faster in the icddr,b SA (Fig. 2). Mortality rates in the age groups 15–64 years were stable in the icddr,b area, but in 2003–2004 in the government SA were little higher and showed a slow declining trend over the years. In the age group 65 years and above, mortality rates were comparable between the two areas and did not show declining trends.

Percentage distribution of deaths and the death rates (per 1,000 person-years) during 2003–2010 by age and area revealed lower neonatal and post-neonatal mortality rates in the icddr,b SA than in the government SA (Table 2). Crude death rate was a little higher in the government SA, mainly due to the higher death rates in the neonatal and post-neonatal periods. Mortality was higher for males than females in the neonatal period and in the age groups 15–49 and 50–64 years in each area.

**Table 2:** Distribution of deaths, death rates^a^ and sex ratios^b^ by age and area, Matlab HDSS 2003–2010

	icddr,b service area			Government service area		
		
Age group	# deaths (%)	Rate^a^	Sex ratio^b^	% (# deaths)	Rate^a^	Sex ratio^b^
0–28 days	485 (8.1)	299.9*	139.8	637 (10.3)	418.9	117.6
29 days to 11 months^c^	161 (2.7)	8.2*	94.4	210 (3.4)	11.4	76.7
1–4 yr	240 (4.0)	2.8	95.7	249 (4.0)	3.0	106.4
5–14 yr	121 (2.0)	0.6	104.9	145 (2.3)	0.7	109.4
15–49 yr	711 (11.9)	1.6	147.4	738 (11.9)	1.7	150.0
50–64yr	989 (16.6)	10.3	183.3	1005 (16.3)	10.8	193.0
65+ yr	3258 (54.6)	59.9	108.3	3196 (51.7)	59.7	121.5
All ages	5965 (100.0)	6.6	132.0	6179 (100.0)	7.0	132.4
# person years	909319			884583		

a per 1000 person years of observation.

b ratio of male to female death rates (100*M/F).

c per 1000 person years in infancy.

* p<0.01(compared between the two areas).

### Difference in cause-specific mortality by area

Mortality rates by broad age- and cause-category showed lower mortality rates due to CDs among children (aged 0–14 years), adults (aged 15–64 years), and elderly in the icddr,b SA than in the government SA, where the mortality rate due to non-NCDs was lower among elderly (Table 3). No death was assigned to the vaccine preventable diseases. Mortality rates due to neonatal causes and pregnancy-related causes in adult females were lower in the icddr,b SA, whereas mortality rates due to external causes were comparable in each age group between these two areas. The rate of indeterminate, however, was higher in the government SA.

**Table 3:** Death rates^a^ by broad age and cause categories in the icddr,b and government service

	Age 0–14 years		Age 15–64 years		Age 65+ years	
			
Disease category	icddr,b	Government	icddr,b	Government	icddr,b	Government
Communicable diseases	0.72	0.99**	0.59	0.74*	10.55	13.55**
Non-communicable diseases	0.34	0.35	1.95	1.87	41.87	38.14**
Neonatal/maternal causes	13.18	1.49**	0.08	0.12$	NA	NA
Injury and external causes	0.58	0.55	0.20	0.28	0.66	0.80
Indeterminate	0.49	0.66**	0.29	0.37	6.59	7.11
All causes	3.31	4.03**	3.29	3.60	59.66	59.60
Person years	303110	306829	551789	524230	54421	53523
Number of deaths	1004	1235	1816	1886	3247	3190

a per 1000 person years of observation;

$ p<0.1

* p<0.05

** p<0.01, (compared between the two areas).

The all-age CSMFs due to CDs were less frequent (18.8% versus 22.9%) in the icddr,b SA, and those due to NCDs were less frequent (50.7% versus 58.2%) in the government SA (Table 4). The most common CDs were acute respiratory infection (ARI)/pneumonia and pulmonary tuberculosis, and these were less frequent in the icddr,b SA. Some NCDs exhibited area differences; stroke (19.9% versus 15.6%), cardiac diseases (8.1% versus 6.1%) and chronic obstetric pulmonary diseases including asthma (6.1% versus 5.1%) were more frequent in the icddr,b SA than the government SA. Malignant neoplasms accounted one in seven deaths (14.2–15.3%), and malignancies were more frequent in the digestive system (5.5–6.0%) followed by the respiratory system (4.1–5.2%) in both areas. Neonatal causes were less frequent (6.0% versus 7.4%) in the icddr,b SA due to less frequent deaths from infections (ARI, pneumonia, or sepsis) and birth asphyxia. Fraction of deaths due to injury and other external causes was comparable (5.4–5.8%) between these two areas. Common external causes were accidental drowning (2.6–2.8%), followed by intentional self-harm (0.7–1.0%).

**Table 4:** Cause-specific mortality fractions (in %) by sex and area, Matlab HDSS 2003–2010

	icddr,b service area			Government service area		
		
Cause of death	Male	Female	Total	Male	Female	Total
Communicable diseases (CDs)	20.5	16.8*	18.8	24.3	21.3*	22.9*
ARI/pneumonia	8.3	8.3	8.3	10.6	10.8	10.7*
Pulmonary tuberculosis	10.0	5.8*	8.1	11.0	6.8*	9.1$
Other CDs	2.3	2.7	2.5	2.7	3.6$	3.1$
Non-communicable diseases (NCDs)	55.9	60.9*	58.2	49.6	52.1$	50.7*
Stroke	15.9	24.6*	19.9	13.9	17.6*	15.6*
Cardiac disease	9.8	6.2*	8.1	7.6	4.4*	6.1*
COPD and asthma	6.1	6.1	6.1	5.3	5.0	5.1*
Malignant neoplasm	16.2	11.8*	14.2	15.9	14.6	15.3
Digestive system	6.1	4.7*	5.5	6.3	5.7	6.0
Respiratory system	5.8	2.2*	4.1	6.1	4.2*	5.2*
Other neoplasm	4.3	4.9	4.6	3.5	4.7$	4.1
Acute abdomen	2.8	4.0*	3.4	2.7	3.3	3.0
Liver cirrhosis	0.5	0.4	0.5	0.6	0.8	0.7
Diabetes mellitus	1.6	2.6*	2.0	1.2	2.0$	1.5$
Anaemia/malnutrition	1.1	2.4*	1.7	1.2	2.4*	1.8
Other NCD	1.2	1.7	1.4	0.7	0.9	0.8*
Perinatal and neonatal causes	6.8	5.0*	6.0	7.5	7.3	7.4*
ARI, pneumonia, sepsis	3.2	2.1*	2.7	3.7	2.8$	3.3$
Birth asphyxia	0.9	0.7	0.8	1.4	1.4	1.4*
Prematurity	1.9	1.6	1.8	1.3	2.1	1.7
Other neonatal cause	0.9	0.6	0.8	1.1	0.9	1.0
Cause related to pregnancy	NA	0.7	0.3	Na	1.0	0.5
Injury and other external cause	5.4	5.5	5.4	6.1	5.4	5.8
Accidental drowning	2.8	2.7	2.8	2.8	2.3	2.6
Road traffic accident	0.7	0.3*	0.5	0.9	0.2*	0.6
International self-harm	0.4	1.2*	0.7	0.5	1.5*	1.0
Assault	0.3	0.5	0.4	0.4	0.5	0.5
Other cause	1.1	0.8	1.0	1.4	1.0	1.2
Indeterminate	11.4	11.0	11.2	12.4	12.8	12.6$
# VA with disease symptom^a^	3217	2729	5,946	3,348	2,819	6,167

Note: ARI=acute respiratory infection; COPD=chronic obstetric pulmonary diseases.

a Excluded from analysis was 129 deaths: 31 cases for no response to VA and 98 cases for no disease symptom or sign prior to death.

$ p<0.05

* p<0.01 (compared between males and females within the areas or between area totals).

### Gender differences in CSMF by area

The distribution of CSMFs by gender shows biosocial differences in mortality risks (Table 4). The frequency of deaths due to CDs was higher among males than females (20.5% versus 16.8% in the icddr,b SA and 24.3% versus 21.3% in the government SA), due mostly to a higher frequency of pulmonary tuberculosis deaths in both areas. Frequencies of ARI/pneumonia and other infections were comparable between males and females. Share of deaths due to NCDs was lower (55.9% versus 60.9% in the icddr,b SA and 49.6% versus 52.1% in the government SA) for males than females. Stroke was more frequent among females than males among whom cardiac diseases were higher in both areas. Frequency of overall malignant neoplasms, particularly neoplasms in respiratory system, was higher among males. CSMFs for acute abdomen, diabetes mellitus, and severe anaemia or malnutrition were relatively low, but significantly higher among females than males in each area.

As expected, the share of neonatal deaths was a little higher (6.8% versus 5.0%) among male deaths than female deaths in the icddr,b SA. The most common neonatal cause was ARI/pneumonia/sepsis. Injury and other external causes were comparable between males and females with half of them being due to accidental drowning, mostly in the age group 1–4 years. Road traffic accident deaths were less common, but higher among males than females, among whom self-harm and assaults were higher.

### Rank-order of CoD by age

Table 5 shows marked variations in the rank-order of top causes of death by age group, as well as the percentage of indeterminate cause. Indeterminate was the highest for neonates (23.2%) and the lowest for children aged 1–4 years (6.8%). Neonatal deaths were due mostly to ARI/pneumonia (26.8%), followed by prematurity (18.6%), birth asphyxia (11.9%), and sepsis (5.5%), summing up to 62.8% of the deaths. In the post-neonatal period, the leading cause was ARI/pneumonia (60.9%), followed by malnutrition (8.5%), diarrhoea (6.3%), meningitis or encephalitis (3.8%), and injury (3.1%), totalling 82.6% of the deaths. Injury and other external cause (49.4%, accidental drowning accounted for 46.4%) were the leading cause of child (age 1–4 years) deaths followed by ARI/pneumonia (16.2%), malnutrition (15.5%), tuberculosis (2.9%), meningitis or encephalitis (2.3%), and diarrhoea (1.7%), totalling 88% of the child deaths. Among older children (age 5–14 years), the leading causes were injury and other external causes (33.5%, accidental drowning accounted for 17.6%), malnutrition (7.4%), acute abdomen (7.4%), ARI/pneumonia (7.1%), neoplasm (5.9%), tuberculosis (5.0%), and meningitis or encephalitis (2.5%); totalling 70.8% of the deaths .

**Table 5:** Cause-specific mortality fractions^a^ [in %] by age group of the deceased in Matlab HDSS, 2003–2010

0–28 days (# deaths=1116)	29 days-<1 year (# deaths=368)	1–4 years (# deaths=489)	5–14 years (# deaths=265)	15–49 years (# deaths=1429)	50–64 years (# deaths=1974)	65+ years (# deaths=6374)
ARI or pneumonia [26.8%]	ARI/pneumonia [60.9%]	Injury [49.4%] Drowning [46.4%]	Injury [33.5%] Drowning [17.6%]	Neoplasm [20.5%]	Neoplasm [22.8%]	Stroke [25.8%]
Prematurity [18.6%]	Malnutrition [8.5%]	ARI/pneumonia [16.2%]	Malnutrition [7.4%]	Injury [14.0%] Self-harm [5.8%]	Stroke [17.3%]	Neoplasm [15.7%]
Birth asphyxia [11.9%]	Diarrhoea [6.3%]	Malnutrition (15.5%)	Acute abdomen [7.4%]	Tuberculosis [11.6%]	Tuberculosis [12.5%]	ARI/pneumonia [9.3%]
Sepsis [5.5%]	Meningitis/encephalitis [3.8%]	Tuberculosis [2.9%]	ARI/pneumonia [7.1%]	Stroke [9.6%]	Cardiac diseases [11.0%]	Tuberculosis [9.2%]
Meningitis/encephalitis [3.3%]	Injury [3.1%]	Meningitis/encephalitis [2.3%]	Neoplasm [5.9%]	Cardiac diseases [7.3%]	COPD/asthma [7.7%]	Cardiac diseases [8.2%]
Malformation [1.2%]	Sepsis [2.2%]	Diarrhoea [1.7%]	Tuberculosis [5.0%]	Acute abdomen [5.5%]	ARI/pneumonia [7.4%]	COPD/asthma [7.6%]
			Meningitis/encephalitis [2.5%]	ARI/pneumonia [5.3%]	Acute abdomen [3.6%]	Acute abdomen [3.1%]
			Epilepsy [2.0]	Maternal cause [3.4%]	Injury [2.8%]	Diabetes [2.7%]
				COPD/asthma [2.3%]	Diabetes [1.6%]	Renal failure [1.2%]
Others [9.4%]	Other [7.3%]	Other [6.4%]	Other [15.2%]	Other [7.7%]	Other [4.2%]	Other [5.6%]
Indeterminate [23.1%]	Indeterminate [7.9%]	Indeterminate [6.8%]	Indeterminate [14.0%]	Indeterminate [12.8%]	Indeterminate [9.1%]	Indeterminate [11.6%]

a Included VA with reported disease symptoms.

The most common CoD among younger adults (age 15–49 years) was malignant neoplasm (20.5%), followed by injury and other external causes (14.0%), tuberculosis (11.6%), stroke (9.6%) and cardiac diseases (7.3%), acute abdomen (5.5%), and ARI/pneumonia (5.3%). These causes accounted for 73.8% of the deaths. Deaths from pregnancy-related causes accounted for 7.5% of the adult female deaths. Malignant neoplasms (22.8%) continued to be the leading cause among older adults (50–64 years), followed by stroke (17.3%), tuberculosis (12.5%), cardiac disease (11.0%), COPD/asthma (7.7%), ARI/ pneumonia (7.4%), acute abdomen (3.6%), and injury (2.8%), accounting for 85.1% of the deaths. The leading causes of elderly (65 years and older) deaths were stroke (25.8%), followed by malignant neoplasm (15.7%), ARI/pneumonia (9.3%), tuberculosis (9.2%), cardiac diseases (8.2%), and COPD or asthma (7.6%), totalling 75.8% of all deaths.

## Discussion

The results showed the differences in health burdens, particularly drop in mortality fractions due to CDs and perinatal and neonatal causes, and a rise in mortality fractions due to NCDs in the icddr,b SA, as compared to the government SA. Earlier studies reported lower infant and child mortality rates in 1982–2002, and lower perinatal and neonatal mortality in 2005–2009 in the icddr,b SA than in the government SA, and the majority of the differences were due to the high quality of MNCH services in the icddr,b area (10, 11). A few studies reported the lower risk of dying from ARI/pneumonia in children under 5 years old in the icddr,b area with enhanced health care system (19, 20). Our results, in support of the previous studies, revealed the simultaneous decrease in mortality from CDs in all ages and increase in mortality rates from NCDs in older ages in the icddr,b SA as compared to the government SA during 2003–2010. This clearly suggests an age shift in mortality towards old age with some compensatory effect on NCDs and on overall death rate in the icddr,b SA, possibly due to the enhanced MNCH-FP services project for a long period. The distribution of the InterVA-4-coded CoDs in broad cause categories was more similar than dissimilar with the physician-coded CoD (6, 21, 22).

The study findings showed a gender difference in mortality fractions due to specific CDs and NCDs, and injuries/external causes in rural areas, indicating a need for differential priorities and public health responses. The finding is consistent with the gender difference found in the distribution of physician-coded CoD of adults and elderly (6). The risk factors of NCDs showed sex difference in the prevalence of hypertension (32% in women and 19% in men aged 35 years and older) in 2011 (21). More than half of the diseased were not aware that they had the disease (21). More men aged 25–64 years used tobacco products (68.2% versus 32.7%) than women of similar ages, who were more often overweight (15.2% versus 10.8%) and more often had self-reported heart problems (10.5% versus 4.9%) (23).

Management of NCDs is available at tertiary-level hospitals, which are very expensive. Many NCDs are, however, amenable to prevention through behaviour changes. Lifestyle and behaviours are linked to 20–25% of the global burden of disease, which is likely to increase rapidly in poorer countries (24). In Bangladesh, use of tobacco products, excessive intake of salt, and abuse of substances are substantially high; and consumption of vegetables and fruits and regular exercise are very low (23). A number of ‘conventional’ NCDs share these same risk factors, and interventions directed towards these will address the diseases simultaneously (25).

Injury and other external causes were the leading cause of mortality in the age groups 1–4 and 5–14 years in this rural community. This finding reconfirmed drowning as the leading CoD for children and recommended undertaking an appropriate intervention for preventing such deaths in rural Bangladesh. An intervention project called ‘Saving lives from drowning’ is currently underway, but the results are yet to be seen. One-fourth (24%) of the deaths in the 15–49 years age group were due to injury and other external causes, half of which were due to self-harm and another 14% were due to assault. Assaults (physical and mental) often provoke self-harm, thus underestimating the share of assault. Violence against women, particularly young women, is quite high in South Asia including Bangladesh and is often perpetrated by the woman's husband or his family members (26–28). Assessment of ‘self-harm and assault’ obtained from family members through VA may underestimate the true burden, therefore indicating a limitation of VA in divulging the true cause in some cases.

The government Health, Population and Nutrition Sector Development Programs (HPNSDPs) for 2011–2016 have an operational plan to control NCDs by expanding access to health services (29). The operation plan of HPNSDP includes conducting training on NCD screening and management for health care providers at district and sub-district levels, organizing awareness-building workshops on injuries, and pilot screening as well as management of selected NCDs at the sub-district level facilities, and gradually expanding to the Union Health and Family Welfare Centre and to the Community Clinic (CC). The private sector, particularly pharmacies in urban and rural areas and ‘workplace based prevention and screening intervention’ can play an important role in screening and referral. Prevention, early detection, and compliance with effective medication can save national health expenditure, improve population health, and reduce household's unnecessary medical costs and loss of productivity. Strengthening behaviour change communications activities at the community level for controlling unhealthy diet and lifestyle and promoting healthy lifestyle and injury prevention may further lower disease and economic burdens. As NCDs bring catastrophic economic consequences and greatly exacerbate poverty, their control must receive due importance to alleviate poverty and improve population health.

In conclusion, the InterVA-4 coded CoD revealed double burdens of CDs and NCDs, with difference in burdens between the two areas and a shift towards NCDs and external causes. The present public health system is focused mainly on infectious diseases that remain of utmost concern, but it needs to be equipped to address the massive burdens of NCDs in rural areas.

## References

[1] United Nations (2013) World population prospects: the 2012 revision, highlights and advance tables. ESA/P/WP.228.

[2] Das P, Horton R (2013). Bangladesh: innovating for health. Lancet.

[3] Sen A (2013). What's happening in Bangladesh?. Lancet.

[4] El AS, Christou A, Reichenbach L, Osman FA, Azad K, Islam KS (2013). Community-based approaches and partnerships: innovations in health-service delivery in Bangladesh. Lancet.

[5] Karar ZA, Alam N, Streatfield PK (2009). Epidemiological transition in rural Bangladesh, 1986–2006. Glob Health Action.

[6] Alam N, Chowdhury HR, Bhuiyan MA, Streatfield PK (2010). Causes of death of adults and elderly and healthcare-seeking before death in rural Bangladesh. J Health Popul Nutr.

[7] Rahman A, Moran A, Pervin J, Rahman A, Rahman M, Yeasmin S (2011). Effectiveness of an integrated approach to reduce perinatal mortality: recent experiences from Matlab, Bangladesh. BMC Public Health.

[8] Rahman M, DaVanzo J, Razzaque A (2010). The role of pregnancy outcomes in the maternal mortality rates of two areas in Matlab, Bangladesh. Int Perspect Sex Reprod Health.

[9] LeGrand TK, Phillips JF (1996). The effects of fertility reductions on infant and child mortality: evidence from Matlab in rural Bangladesh. Popul Stud.

[10] Pervin J, Moran A, Rahman M, Razzaque A, Sibley L, Streatfield PK (2012). Association of antenatal care with facility delivery and perinatal survival – a population-based study in Bangladesh. BMC Pregnancy and Childbirth.

[11] Hale L, DaVanzo J, Razzaque A, Rahman M (2006). Why are infant and child mortality rates lower in the MCH-FP Area of Matlab, Bangladesh?. Stud Fam Plann.

[12] Soleman N, Chandramohan D, Shibuya K (2006). Verbal autopsy: current practices and challenges. Bull World Health Organ.

[13] Byass P (2007). Who needs cause-of-death data?. PLoS Med.

[14] Murray CJ, Lozano R, Flaxman AD, Vahdatpour A, Lopez AD (2011). Robust metrics for assessing the performance of different verbal autopsy cause assignment methods in validation studies. Popul Health Metr.

[15] Byass P, Chandramohan D, Clark SJ, D'Ambruoso L, Fottrell E, Graham WJ (2012). Strengthening standardised interpretation of verbal autopsy data: the new InterVA-4 tool. Glob Health Action.

[16] James SL, Flaxman AD, Murray CJ (2011). Performance of the Tariff Method: validation of a simple additive algorithm for analysis of verbal autopsies. Popul Health Metr.

[17] Leitao J, Chandramohan D, Byass P, Jakob R, Bundhamcharoen K (2013). Revising the WHO verbal autopsy instrument to facilitate routine cause-of-death monitoring. Glob Health Action.

[18] INDEPTH Network (2014). INDEPTH Network Cause-Specific Mortality – Release 2014. http:\\www.indepth-network.org.

[19] Ali M, Emch M, Tofail F, Baqui AH (2001). Implications of health care provision on acute lower respiratory infection mortality in Bangladeshi children. Soc Sci Med.

[20] Fauveau V, Stewart MK, Chakraborty J, Khan SA (1992). Impact on mortality of a community-based programme to control acute lower respiratory tract infections. Bull World Health Organ.

[21] BDHS (2012). Chapter 14: Cause of death in children under age 5. Bangladesh Demographic and Health Survey 2011.

[22] BMMS (2011). BMMS 2010: Chapter 3: Adult female mortality: levels and causes. Bangladesh Maternal Mortality and Health Care Survey 2010.

[23] Ahmed SM, Hadi A, Razzaque A, Ashraf A, Juvekar S, Ng N (2009). Clustering of chronic non-communicable disease risk factors among selected Asian populations: levels and determinants. Glob Health Action.

[24] WHO (2008). 2008–2013 action plan for the global strategy for the prevention and control of non-communicable diseases. http://whqlibdoc.who.int/publications/2009/9789241597418_eng.pdf.

[25] Lawes CM, Vander HS, Rodgers A (2008). Global burden of blood-pressure-related disease, 2001. Lancet.

[26] Bates LM, Schuler SR, Islam F, Islam K (2004). Socioeconomic factors and processes associated with domestic violence in rural Bangladesh. Int Fam Plan Perspect.

[27] Koenig MA, Ahmed S, Hossain MB, Khorshed AMAB (2003). Women's status and domestic violence in rural Bangladesh: individual- and community-level effects. Demography.

[28] WHO (2003). WHO 2003 – Multi-country Study on Women's Health and Domestic Violence against Women.

[29] Ministry of Health and Family Welfare, Planning Wing MoHaFW, Government of the People's Republic of Bangladesh (2011). HPNSDP (Health, Population and Nutrition Sector Development Program) 2011–2016: Program Implementation Plan.

